# High prevalence of morphological subtype FAB M1 in adults with *de novo* acute myeloid leukemia in São José dos Campos, São Paulo

**DOI:** 10.1590/S1516-31802006000100010

**Published:** 2006-01-05

**Authors:** Fernando Callera, Carla Cecília Mulin, Evandro Secchi Rosa, Djanete Barbosa de Melo, Cláudio Marcelo Tavares Pessoa de Melo

**Keywords:** Acute myelogenous leukemia, Leukemia, Prevalence, Neoplasms, Diagnosis, Leucemia mielocítica aguda, Leucemia, Prevalência, Câncer, Diagnóstico

## Abstract

**CONTEXT AND OBJECTIVE:**

Geographical variations have been described in acute myelogenous leukemia (AML). In Brazil, few studies have been published on this. The aim of this study was to demonstrate the high prevalence of French- American-British (FAB) M1 subtype in adults with *de novo* AML in São José dos Campos, State of São Paulo, Brazil.

**DESIGN AND SETTING:**

Retrospective analysis, at Hospital Pio XII in São José dos Campos, a public non-teaching institution.

**METHODS:**

Records from 39 consecutive adult patients with *de novo* AML referred to Hospital Pio XII between January 2002 and September 2004 were reviewed. Peripheral blood and blood marrow smears were reviewed blindly by five hematologists and classified according to FAB criteria. The rates of remission, relapse, mortality according treatment phase, survival and leukemia-free survival were calculated.

**RESULTS:**

The prevalence of each category as determined via a consensus among five observers was M0: 0%; M1: 43.6%; M2: 30.7%; M3: 12.8%; M4: 5.1%; M5: 2.6%: M6: 2.6%; and M7: 2.6%. The remission and the relapse rates were 82% and 41% respectively. The mortality rate was 69% (induction of remission: 7/39, 17.9%; post induction: 10/32, 31.2%; and relapse: 10/16, 62.5%). The survival rate was 30% and leukemia-free survival was 33%.

**CONCLUSIONS:**

The study demonstrated a high prevalence of FAB M1 subtype in adults with *de novo* AML in São José dos Campos. Our data suggest the occurrence of different regional prevalences of FAB AML categories in Brazil.

## RESUMO

**CONTEXTO E OBJETIVO:**

Variações geográficas têm sido demonstradas na leucemia mielóide aguda (LMA). No Brasil poucos estudos têm sido publicados nesta área. O objetivo foi demonstrar a alta prevalência do subtipo FAB M1 em adultos com LMA *de novo* em São José dos Campos, São Paulo.

**TIPO DE ESTUDO E LOCAL:**

Análise retrospectiva, no Hospital Pio XII de São José dos Campos, instituição pública e não ligada a universidade.

**MÉTODOS:**

Registros de 39 pacientes consecutivos com LMA *de novo* foram analisados de janeiro de 2002 até setembro de 2004. Lâminas de sangue periférico e da medula óssea sem identificação prévia foram analisadas por cinco hematologistas e classificadas de acordo com os critérios FAB. Taxas de remissão, de recaída, de mortalidade, de mortalidade de acordo com o período de tratamento, de sobrevivência e de sobrevivência livre de leucemia foram calculadas.

**RESULTADOS:**

A prevalência de cada categoria determinada por consenso entre os observadores foi M0: 0, M1: 43,6%, M2: 30,7%, M3: 12,8%, M4: -5,1%, M5: 2,6%, M6: 2,6% and M7: 2,6%. As taxas de remissão e de recaída foram de 82% e 41% respectivamente. A taxa de mortalidade foi de 69% (indução da remissão 7/39, 17.9%, pós-indução 10/32, 31,2% e de recaída 10/16, 62,5%). A taxa de sobrevivência foi de 30% e a taxa de sobrevivência livre de leucemia foi de 33%.

**CONCLUSÕES:**

O estudo demonstrou alta prevalência do subtipo FAB M1 em adultos com LMA *de novo* em São José dos Campos. Nossos resultados sugerem a ocorrência de diferentes prevalências de subtipos FAB nas regiões do Brasil.

## INTRODUCTION

Geographical heterogeneity of morphological categories defined by the French-American-British (FAB) criteria has been demonstrated in acute myelogenous leukemia (AML). High prevalence of FAB M3 subtype has been demonstrated in Brazilian patients. Moreover, there are regions in Brazil such as Campinas (State of São Paulo) and Teresina (State of Piauí) where the most common subtypes are M4 and M2 respectively. FAB M2 has been demonstrated to be the most frequent subtype in Japanese patients and M4 in patients from Australia.^1-4^

The FAB M1 subtype accounts for less than 20% of the FAB categories and it has been more frequently described in young adults.^5^ In clinical practice, we have noticed an apparently high proportion of AML M1 subtype in older patients in São José dos Campos, State of São Paulo, Brazil.

## OBJECTIVE

The aim of this study was to demonstrate the high proportion of morphological FAB M1 subtype in adults with *de novo* AML in São José dos Campos.

## METHODS

### Type of study:

retrospective analysis.

### Setting:

Patients were isolated in single or double bedrooms at Hospital Pio XII, a public non-teaching hospital in São José dos Campos that provides a full range of medical and surgical services including treatment for hematological malignancy and autologous bone marrow transplantation. AML patients from the 21^st^ Regional Health Division of the State of São Paulo, which is composed of twelve municipalities in the Vale do Paraíba region (over 1,000,000 inhabitants), are referred to Hospital Pio XII.

### Sample:

Records from 39 consecutive adult patients (> 18 years old) with *de novo* AML attended between January 2002 and September 2004 were reviewed. No one was excluded.

### Diagnosis:

The diagnosis of *de novo* AML was confirmed by means of cytological examination of peripheral blood (PB) and bone marrow (BM) and classified according to the FAB criteria.^5^ PB and BM smears were reviewed blindly by five hematologists. The rate of diagnostic concordance among the observers was 78%. Discrepancies were reviewed once again and each FAB category was determined via a consensus among the five observers.

### Chemotherapy:

All patients were treated according to the current standard induction regimen.^1,2^

### Blood Banking:

There was no lack of blood components during the study.

### Antimicrobial agents:

We followed the 2002 guidelines for the use of antimicrobial agents in neutropenic patients with cancer and there was no lack of antimicrobial agents during the study.

### Remission and relapse criteria:

Complete remission was defined as M1 marrow status (< 5% blasts; > 15% erythroid elements; and > 25% normal granulocyte precursors). Relapse was defined as the appearance of circulating leukemia cells or > 5% blasts in the BM.

### Main measurements:

The incidence of FAB categories, the remission rate, the relapse rate, the mortality rate according to treatment phase, the survival rate and the leukemia-free survival rate were measured.

### Statistical methods:

The survival rate and leukemia-free survival rate were calculated by means of the Kaplan Meier method (SPSS software, SPSS Inc., Chicago, United States).

### Consent:

This study was authorized by the ethical committee of Hospital Pio XII.

## RESULTS

The clinical and laboratory characteristics of the 39 patients with AML are shown in Table 1, with predominance (43.6%) of M1 FAB classification. Immunophenotyping was performed on 20 patients (51%), using markers for the clusters of differentiation (CD) CD13 and CD33 and myeloperoxidase-positive markers. There was an association with CD7 in three patients and human leukocyte antigen DR was negative in three patients with FAB M3 subtype. Karyotyping was performed on ten patients (25.6%); cytogenetic abnormalities were observed in seven: one with normal karyotype, one with complex karyotype, two with t (15;17), one with t (8;21) and two with trisomy 8.

**Table 1:** Clinical and laboratory characteristics of the 39 patients with *de novo* acute myelogenous leukemia treated in São José dos Campos between January 2002 and September 2004

Characteristics	Quantity
Sex: number (%)	
Male	22 (56)
Female	17 (44)
Male-to-female ratio	1.3:1
Age (years)	
Median	52
Range	18-84
Age group: number (%)	
< 35 years	8 (20.5)
35-60 years	17 (43.5)
> 60 years	14 (36)
Performance status: Karnovsky (%)	
Median	70
Range	50-100
White-cell count (x 10^3^/mm^3^)	
Median	6.4
Range	0.4-344
Hemoglobin (g/dl)	
Median	8.4
Range	5.0-12.4
Platelet count (x10^3^/mm^3^)	
Median	44
Range	12-240
Peripheral blast count (%)	
Median	39
Range	0-100
Bone marrow blast count (%)	
Median	100
Range	21-100
FAB classification: number (%)	
M0	0 (0)
M1	17 (43.6)
M2	12 (30.7)
M3	5 (12.8)
M4	2 (5.1)
M5	1 (2.6)
M6	1 (2.6)
M7	1 (2.6)

*FAB = French-American-British.*

Overall, 32 patients (82%) achieved remission; 16 (41%) relapsed within one year after their diagnosis. Twenty-seven patients (69%) died and most of them did so within six months after their diagnosis (Table 2). The survival rate was 30% and the leukemia-free survival rate was 33%.

**Table 2:** Clinical characteristics of the 27 patients with acute myeloid leukemia, treated in Hospital Pio XII, who died

Characteristic	Number (%)
Deaths	
Male	18 (66.7)
Female	9 (33.3)
Total	27/39 (69)
Age	(years)
Median	54
Range	18-84
Age group	
< 35 years	5/8 (62.5)
35-60 years	10/17(58.8)
> 60 years	12/14 (85.7)
Deaths according to the treatment phase or relapse	
Induction of remission	7/39 (17.9)
Post-induction	10/32 (31.2)
Relapse	10/16 (62.5)
Induction of remission and systems associated with mortality	
Respiratory	3
Hemostatic (disseminated intravascular coagulation)	2
Nervous (tumor infiltration)	2
Post-induction and systems associated with mortality	
Respiratory/hemostatic (disseminated intravascular coagulation)	3
Multiple organ failure	3
Respiratory/cardiovascular	2
Respiratory/renal	1
Nervous (tumor infiltration)	1
Relapse and systems associated with mortality	
Multiple organ failure	4
Respiratory/hemostatic (disseminated intravascular coagulation)	3
Respiratory/renal	1
Hemostatic (disseminated intravascular coagulation)	1
Renal	1

## DISCUSSION

The high prevalence of the FAB M1 subtype in this group of patients is not similar to what has previously been described.^1-4^ There are some FAB categories such as M3 AML that are so distinctive that there is a high concordance rate among observers. Problems arise in distinguishing between M1 and M2 and between M2 and M4 AML. Overall, the rates of concordance have varied from 58 to 98%, thus reinforcing the understanding that the accuracy of categorization depends on the skill and experience of the observers.^5^ Therefore, the present cases were reviewed blindly by five hematology morphologists who already had expertise in this field. By using this method we attempted to decrease the element of arbitrariness, decrease the likelihood of misdiagnosis and improve the accuracy of classification. We achieved concordance between all the observers in 78% of the cases, thus confirming that the categorization of AML subtypes by means of the FAB criteria is reliable and reproducible. It should be noted that agreement was not achieved in the cases of nine patients. The BM smears of these particular cases were reviewed once again. The purpose of this procedure was to improve the classification accuracy and to achieve a consensus among the five observers.

Nonetheless, the high proportion of FAB M1 subtype was based on a sample obtained from a single institution and it should be emphasized that the number of patients studied may be not representative of the entire region. Similar problems have been observed in other studies.^1,2^ Considering the incidence rate of new cases of AML in adults in relation to the number of inhabitants in this region (over 1,000,000 inhabitants) and the period of time studied, a reasonable number of patients were studied. However, further cooperative studies enrolling a greater number of patients would be necessary in order to clarify this point.

Finally, the remission, relapse and mortality rates observed during the induction of remission and during the post-induction period, the survival rate and the leukemia-free survival rate were similar to what has been observed in other regions of Brazil.^1-3^

## CONCLUSIONS

We have demonstrated that there is high prevalence of the FAB M1 morphological subtype in adults with *de novo* AML in São José dos Campos. Our data suggest that different prevalences of FAB AML categories occur in different regions of Brazil.
